# Oviposition site selection by *Gasterophilus pecorum* (Diptera: Gasterophilidae) in its habitat in Kalamaili Nature Reserve, Xinjiang, China

**DOI:** 10.1051/parasite/2015034

**Published:** 2015-11-30

**Authors:** Shan-Hui Liu, De-Fu Hu, Kai Li

**Affiliations:** 1 Key Laboratory of Non-invasive Research Technology for Endangered Species, College of Nature Conservation, Beijing Forestry University 100083 Beijing P.R. China

**Keywords:** Przewalski’s horses, Oviposition site, *Gasterophilus pecorum*, Kalamaili Nature Reserve

## Abstract

Oviposition site selection is an important aspect of the behavioural ecology of insects. A comparison of the habitats used by a species enhances our understanding of their adaptation to altered environments. We collected data on the oviposition behaviours of *Gasterophilus pecorum* (Diptera: Gasterophilidae) in its habitat in Kalamaili Nature Reserve (KNR), Xinjiang, China between March and October 2014. We found 91 quadrats were used by *G*. *pecorum* for oviposition. Examining 13 ecological factors using the *t-*test, chi-square test, and principal component analysis showed that *G*. *pecorum*’s oviposition habitat was preferentially on slopes with inclinations of 10–30° that were semi-sunny, semi-cloudy slopes, in positions high or low on the slopes, with preferences for total plants lower than 10% and *Stipa capillata* coverage lower than 10% on the low slopes, but *Ceratoides latens* coverage on the high and intermediate slopes, when the numbers of plant species and families were lower than five. *G*. *pecorum* often selected sites at a distance < 2000 m from a water source and average altitude 900–1000 m. The oviposition site selection by *G*. *pecorum* may be correlated with the behaviour of Przewalski’s horses (*Equus ferus przewalskii*), and water and food resources may strongly influence oviposition site selection, as Przewalski’s horses rest and forage in these areas.

## Abstract (fr)

Le choix du site de ponte est un aspect important de l’écologie comportementale des insectes. Une comparaison des habitats utilisés par une espèce améliore notre compréhension de son adaptation à des environnements modifiés. Nous avons recueilli des données sur les comportements de ponte de *Gasterophilus pecorum* (Diptera, Gasterophilidae) dans son habitat dans la Réserve Naturelle Kalamaili (KNR), Xinjiang, Chine, entre mars et Octobre 2014. Nous avons trouvé 91 quadrats qui étaient utilisés par *G. pecorum* pour la ponte. Un examen de treize facteurs écologiques en utilisant le test *t*, le test du chi carré et une analyse en composantes principales a montré que l’habitat de ponte de *G. pecorum* était préférentiellement des pentes avec des inclinaisons de 10–30° qui étaient semi-ensoleillées, semi-nuageuses, dans des positions haute ou basse sur les pentes, avec des préférences pour une couverture totale par les plantes inférieure à 10 % et inférieure à 10 % pour *Sipa capillata* sur les pentes basses, et pour une couverture par *Ceratoides latens* sur les pentes hautes et intermédiaires où le nombre d’espèces et de familles de plantes étaient inférieur à cinq. *Gasterophilus pecorum* sélectionne souvent des sites à une distance inférieure à 2000 m d’une source d’eau et une altitude moyenne de 900–1000 m. Le choix du site de ponte par *G. pecorum* peut être corrélé avec le comportement des chevaux de Prjevalski (*Equus ferus przewalskii*), et les ressources en eau et de nourriture peuvent influer fortement sur le choix du site de ponte, car les chevaux de Prjevalski se reposent et paissent dans ces zones.

## Introduction

Horses and other equines are hosts to the larvae of the *Gasterophilus* species causing gastrointestinal myiasis [23]. They are prone to *Gasterophilus* infections because the pastures where they graze are contaminated with infective stages (eggs and larvae) of *Gasterophilus*, resulting in continuous infestation and re-infestation. Parasites can have various effects on the health [43, 47, 51, 60] and behaviour [26, 48] of their hosts. Several carefully controlled studies suggest that host mortality rates increase with parasite burden, while the fecundity of infested hosts decreases significantly [30]. Therefore, it is important to examine the ecological factors that determine parasite loads, including environmental factors (climate, humidity, temperature, rainfall, vegetation, stocking density, and management) [41, 44], and the feeding, movement, and defecation patterns of the host, which determine the parasites encountered [26, 31]. Przewalski’s horse, which was listed as endangered by the International Union for Conservation of Nature in 2011, was once distributed widely in the Eurasian desert steppe; however, it became extinct in the wild in the middle of the last century [5, 35], with a few remnant populations existing as small captive breeding herds in western countries [29]. Efforts to reintroduce Przewalski’s horse in Central Asia, Mongolia, Russia, and north-western China started in the 1990’s [29]. In northwest China, Przewalski’s horses were released into the Kalamaili Nature Reserve (KNR) in Xinjiang in August 2001, and the population has subsequently increased significantly [28, 30, 59].

Movement strategies are crucial for the survival of animals that need to find resources or escape from predators or parasites [34, 54]. *Gasterophilus pecorum* is the dominant botfly species and aggressively attacks horses in the KNR and accounts for the vast majority of all parasites infecting them [28, 30, 34]. In addition, the oviposition sites of *G*. *pecorum* differ from those of other botflies. *G*. *pecorum* mainly lays its eggs on grass, while other species lay their eggs on horse hair, forelegs, lips, face and the intermandibular area [61]. Although deworming is performed annually during the winter in these Przewalski’s horses, recurring *Gasterophilus* infections remain prevalent and the parasitic burden is severe with an infection rate of 100% [30]. *Gasterophilus pecorum* was the dominant species and was found to aggressively attack equids in the KNR and to account for the vast majority of all parasites infecting equids [30]. In contrast, research reports that *Gasterophilus intestinalis* and *Gasterophilus nasalis* are the dominant species in other regions of the world [38, 61]. What has caused this? Where are the oviposition sites of *G*. *pecorum*? No study has examined this so far. The distribution of water [46] and food resources [18] has a major impact on the movement patterns of many large herbivores, particularly equids. The movement of host animals may also affect *Gasterophilus* life cycles. The distribution of both host and parasite is crucial, as the population sizes and their interconnections determine co-evolutionary outcomes [4, 10]. The spatial distributions and co-evolution of parasites and hosts usually overlap completely [6]. Research on parasitological examinations in the three equid species shows how the factors home range, social structure, and resource selection, significantly impact parasitic burden [39]. Species of *Gasterophilus* are obligate parasites of animals like horses, donkeys, and mules in their larvae stage of the life cycle [55]. A large number of eggs can be laid by the female fly, and the eggs of various species differ in colour and laying location in the host body. In *G. pecorum* the eggs are laid in batches of 10–115 and distributed on pasture vegetation. The eggs are easily observable and hatch spontaneously in about 3–9 days. In *G. pecorum* eggs hatch within 3 to 5 min in the mouth [60].

During warm months, Przewalski’s horses move to high, bare places where there is no forage during the warmer hours of the day. They likely move to escape flies, although the flies tend to follow [26]. *G. pecorum* has distribution in Europe, Africa, and Asia, and its common names are botflies and dark-winged horse bot [60]. Large numbers of attached larvae can cause inflammation, hinder swallowing, and may eventually lead to death resulting from constriction of the oesophagus [55]. In Mongolia, only one fly belonging to *Gasterophilus* was found [26], and at times none were observed [39]. The reason for the differences in the level of infection between China and Mongolia remains unknown. It is possible that the level of *G. pecorum* infection in Przewalski’s horses is associated with the unique geographic environment [57]. The features of natural conditions and biological combinations are extremely arid and semi-arid desert in KNR. KNR and Tashikuergan nature reserve in Xinjiang with desert plateau characteristics are typical of similar kinds of areas in China and elsewhere in the world. This position was the ancient ecological corridor for the Eurasian continent and is the ancestral home of Przewalski’s horses.

When studying the effects of changes in host numbers and population structure on disease spread and persistence, one needs to consider variation in the life histories of the parasites [19]. The selection of oviposition habitat evolves to ensure parasite reproduction. Adult *Gasterophilus* spp. lay eggs during specific seasons of the year in most regions of the world [15]. Some monitored studies show egg-laying activity with a bimodal trend with a higher first peak in October–November that decreases suddenly from December to January, although different breeding conditions can significantly affect the average number of eggs laid [41]. Many studies have examined egg laying in botflies, most have focused on when the eggs are laid and the relationship between oviposition and local climate [52]. To our knowledge, some studies have examined the specific location of oviposition in *Gasterophilus* [15, 41]. However, these results did not include *G. pecorum* and did not describe the egg position and habitat features in detail, especially the specific geographical environment in China’s Xinjiang where three kinds of sympatric equines live. Therefore, this study first examined *G*. *pecorum* oviposition habitat selection, and then compared it with wild horse habitat selection and behaviour, to identify why *G*. *pecorum* has become the dominant botfly species.

## Materials and methods

### Study site

The research site was situated in KNR in Xinjiang Province, China (44°40′ to 46°0′ N latitude, 88°33′ to 90°0′ E longitude). KNR consists mainly of the Gobi Desert and semi-desert at altitudes of 500–1200 m. It has a typical continental climate with an average annual temperature of 0.2 °C (range −49.7 to 45.3 °C) and annual precipitation of less than 200 mm, with a 6-month winter [12].

There are four dominant plant species in KNR: *Haloxylon ammodendron*, *Reaumuria soongorica*, *Ceratoides latens*, and *Anabasis salsa* [33]. There are 21 water sources: 13 permanent and eight seasonal [58]. KNR is one of the few places in the world where the habitats of different wild equids overlap: Przewalski’s horse (*Equus ferus przewalskii*), the Mongolian wild ass (*Equus hemionus hemionus*), and domestic horse (*Equus ferus caballus*) live sympatrically and share pastures seasonally. During the study period, the population of free-ranging Przewalski’s horses ranged from 86 to 102 horses in six to nine harems and two bachelor groups [58]. Typically, groups of Przewalski’s horses consist of one adult male, several adult females, and their immature offspring, with group sizes of 4–13 individuals [29].

### Habitat observations and *Gasterophilus* sampling

We observed two groups of Przewalski’s horses in KNR between March and October 2014. We recorded the location of the groups on a 1:10,000 scale map at 15-min intervals and used the *ad libitum* sampling method [1, 2] to record when the horses entered foraging and resting sites and any behaviours and intergroup encounters. After the wild horses left, we set a 10 × 10 m quadrat as focal quadrat for determining habitat factors when the eggs of *G. pecorum* were found [10, 29]. We also measured the food selection ratio for different food species in the diets of each focal group [29]. A contrast quadrat was placed within about 100 m of the focal quadrat and the same habitat factors were measured. A total of 91 oviposition sites of *G. pecorum* and 90 contrast quadrats were marked on the map. For each oviposition site, we recorded 13 habitat factors (see Table 1 for descriptions and survey methods). For vegetation type, slope gradient, slope direction, slope position, and elevation, the percentage of a particular category within the home range of the horse was considered the availability area.

**Table 1.** Frequency distribution of habitat factors in oviposition site selection by *G*. *pecorum* in different habitats in KNR.

Habitat factor	Category	Q_C_	Q_O_	Q_T_	(Q_O_/Q_T_)	*n*	∑(Q_O_/Q_T_)
Altitude (m)	<**900**	1	0	1	0	3	0.57
	900–1000	68	91	159	0.57	3	0.57
	1000–1100	22	0	22	0	3	0.57
Total vegetation coverage (%)	<10	3	0	3	0	4	1.25
	10–20	15	5	20	0.25	4	1.25
	20–30	62	73	135	0.54	4	1.25
	>30	10	13	23	0.56	4	1.25
*Stipa capillata* coverage (%)	<10	5	10	15	0.67	4	1.634
	10–20	18	72	90	0.8	4	1.63
	20–30	52	9	61	0.15	4	1.63
	>30	15	0	15	0	4	1.634
*Stipa capillata* frequency (%)	<**40**	28	19	47	0.40	3	1.49
	40–60	45	50	95	0.53	3	1.49
	>60	17	22	39	0.56	3	1.49
*Stipa capillata* height (cm)	<**10**	31	26	57	0.46	3	1.44
	10–20	37	50	87	0.57	3	1.44
	>20	22	15	37	0.41	3	1.44
*Ceratoides latens* coverage (%)	<**5**	3	4	7	0.57	3	1.27
	5–10	37	64	101	0.63	3	1.27
	>10	50	23	73	0.32	3	1.27
*Artemisia* sp. coverage (%)	<**5**	13	42	55	0.76	3	1.39
	5–10	49	46	95	0.48	3	1.39
	>10	28	3	31	0.097	3	1.39
Vegetation families	<5	78	82	160	0.51	3	1.29
	5–10	10	8	18	0.44	3	1.29
	>10	2	1	3	0.33	3	1.29
Vegetation species	<5	79	77	156	0.49	3	1.34
	5–10	9	13	22	0.59	3	1.34
	>10	2	1	3	0.33	3	1.34
Distance to nearest water (m)	<2000	70	77	147	0.52	3	0.92
	2000–5000	20	14	34	0.41	3	0.92
	>5000	0	0	0	0	3	0.92
Distance to nearest path (m)	<30	65	71	136	0.52	3	0.92
	30–60	13	16	29	0.55	3	0.92
	>60	12	4	16	0.25	3	0.92
Slope direction	S_S_	13	12	25	0.48	4	1.91
	S_C_	8	3	11	0.27	4	1.91
	S_1/2_	5	9	14	0.64	4	1.91
	S_ϕ_	64	67	131	0.51	4	1.91
Slope position	P_U_	31	29	60	0.48	4	2.20
	P_I_	6	10	16	0.62	4	2.20
	P_L_	2	3	5	0.6	4	2.20
	P_ϕ_	51	49	100	0.49	4	2.20
Slope gradient (°)	<10	70	68	138	0.49	4	1.48
	10–20	15	21	36	0.58	4	1.48
	20–30	3	2	5	0.4	4	1.48
	>30	2	0	2	0	4	1.48

Abbreviations: Q_C_, contrast quadrat; Q_O_, oviposition site quadrat; Q_T_, total quadrats; *n*, eigenvalue; P_U_, upper position, located in the upper 1/3 of the slope; P_I_, intermediate position, located in the central part of the slope; P_L_, lower position, located in the lower 1/3 of the slope; P_ϕ_, no slope position; S_1/2_, semi-sunny, semi-cloudy slope, i.e., N 22.50° ~ E S67.5° E, S22.5° W ~ N67.5° W; S_S_, sunny slope, S67.5° ~ E S22.50° W; S_C_, cloudy slope, S67.5° W ~ N 22.5° E; S_ϕ_, no slope direction.

### Data analysis

To test for selection or avoidance of a particular category, Ivlev’s electivity index, *Ei*, was calculated as *Ei* = (*Wi –* 1/*n*)/(*Wi +* 1/*n*) and *Wi* was calculated as *Wi* = (Q_O_/Q_T_)/∑(Q_O_/Q_T_), where *Ei* and *Wi* are respectively choose coefficient and selection index to measure whether or not species choose a particular habitat. In addition, where Q_O_ is the mean proportion of observations in a particular category, Q_T_ is the mean proportion of this category in the home range, *i* is the mean characteristic value, and *n* is the mean eigenvalue of the ecological factors [13, 27, 29]. Then, we used Spearman’s rank correlation to examine correlations between the locations of oviposition sites and the occurrence of seasonal horse behaviour. We used one-way analysis of variance (ANOVA) to test seasonality effects on the usage of oviposition sites. PCA techniques for the identification of common factors in data were used to analyse the main components on adult *Gasterophilus* spp. lay eggs [9]. All data were analysed using SPSS 20.0.

## Results

### Frequency distribution and selection of habitat factors

**Figure 1.**
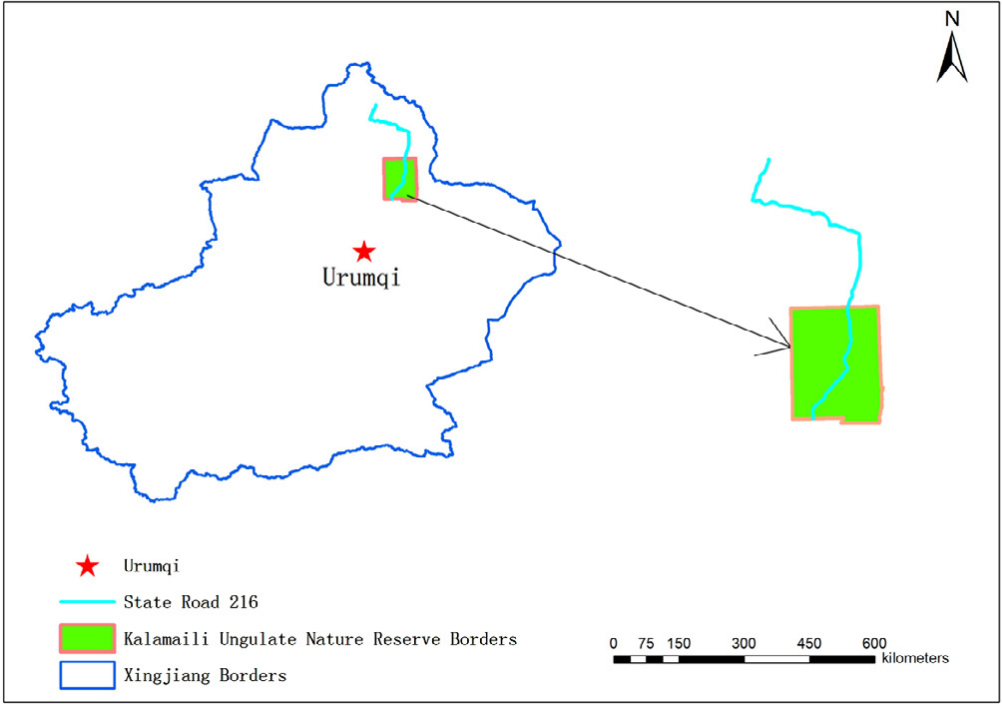
Location of the Kalamaili Nature Reserve in Xingjiang, China.

The two horse groups used seasonal and permanent water sources (Fig. 1). The greatest distance from an oviposition site to the nearest water source was <500 m (46/91). Table 2 shows that *G*. *pecorum* preferred semi-sunny and semi-cloudy slopes, higher and lower slope positions, and slopes of 10–30°. It preferred total plants < 10% and *Stipa capillata* coverage < 10% in the lower slopes, but *Ceratoides latens* coverage in higher and intermediate slopes. In the lower slope habitat, the numbers of plant species and families was < 5. Oviposition sites were often near a water source with a distance < 2000 m and average altitude of 900–1000 m. Table 2 presents the frequency distribution of different habitat factors. Most oviposition sites (66/91) were found within 20 m of the nearest path used by equids.

**Table 2.** Selective analysis and electivity index of habitat factors in oviposition site selection by *G*. *pecorum* in summer in KNR.

Habitat factor	Category	Wi	Ei	Result
Altitude (m)	<**900**	0	−1.0	UC
	900–1000	1	0.5	C
	1000–1100	0	−1.0	UC
Total vegetation coverage (%)	<10	0	−1.0	UC
	10–20	20	−0.1	UC
	20–30	0.45	0.3	C
	>30	0.36	0.2	C
*Stipa capillata* coverage (%)	<10	0.10	−0.4	UC
	10–20	0.34	0.2	C
	20–30	0.32	0.1	C
	>30	0.23	0.0	R
*Stipa capillata* frequency (%)	<**40**	0.27	−0.1	UC
	40–60	0.35	0.0	R
	>60	0.38	0.1	C
*Stipa capillata* height (cm)	<**10**	0.32	0.0	R
	10–20	0.40	0.1	C
	>20	0.28	−0.1	UC
*Ceratoides latens* coverage (%)	<**5**	0.20	−0.3	UC
	5–10	0.42	0.1	C
	>10	0.39	0.1	C
*Artemisia* sp. coverage (%)	<**5**	0.53	0.2	C
	5–10	0.36	0.0	R
	>10	0.11	−0.5	UC
Vegetation families	<5	0.40	0.1	C
	5–10	0.34	0.0	R
	>10	0.26	−0.1	UC
Vegetation number	<5	0.40	0.1	C
	5–10	0.23	−0.2	UC
	>10	0.25	−0.1	UC
Distance to nearest water (m)	<2000	0.57	0.3	C
	2000–5000	0.43	0.1	C
	>5000	0	−1.0	UC
Distance to nearest path (m)	<30	0.57	0.3	C
	30–60	0.60	0.3	C
	>60	0.27	−0.1	UC
Slope direction	S_S_	0.25	0.0	R
	S_C_	0.14	−0.3	UC
	S_1/2_	0.34	0.1	C
	S_ϕ_	0.27	0.0	R
Slope position	P_U_	0.22	−0.1	UC
	P_I_	0.28	0.1	C
	P_L_	0.27	0.0	R
	P_ϕ_	0.22	−0.1	UC
Slope gradient (°)	<10	0.33	0.1	C
	10–20	0.40	0.2	C
	20–30	0.27	0.0	R
	>30	0	−1.0	UC

Abbreviations: UC, not chosen; C, chosen; R, random; other abbreviations as in Table 1.

In the principal component analysis of habitat factor on oviposition site quadrats, the main factors were coverage, food, distance, terrain, and altitude (Table 3).

**Table 3.** PCA variable loadings among the oviposition site selection by *G*. *pecorum* associated factors.

Factors	Axis 1	Axis 2	Axis 3	Axis 4
Altitude (m)	−0.007	0.005	0.004	−0.003
Total vegetation coverage (%)	0.027	−0.031	−0.359	0.114
*Stipa capillata* coverage (%)	0.017	−0.060	−0.396	0.130
*Stipa capillata* frequency (%)	−0.040	−0.132	0.651	0.401
*Stipa capillata* height (cm)	−0.017	0.040	−0.312	0.700
*Ceratoides latens* coverage (%)	0.022	0.102	−0.187	0.157
*Artemisia* sp. coverage (%)	0.017	−0.016	−0.170	0.282
Vegetation families	−0.001	−0.090	0.054	0.147
Vegetation number	0.005	−0.055	0.073	0.091
Distance to nearest water (m)	−0.003	−0.036	−0.110	−0.119
Distance to nearest path (m)	−0.066	0.039	0.307	0.354
Slope direction	−0.025	−0.105	−0.075	0.203
Slope position	0.996	0.002	0.062	0.043
Slope gradient (°)	−0.006	0.970	0.067	0.047
Eigenvalues	1.913	1.168	0.611	0.530
Percentage	32.572	19.886	10.408	9.025
Cumulative percentage	32.572	52.458	62.866	71.892

Abbreviations: Principal components analysis (PCA): PCA is a mathematical procedure that uses an orthogonal transformation to convert a set of observations of possibly correlated variables into a set of values of uncorrelated variables called principal components. Axis: Analysing the characteristic of the main ingredients of core vector in PCA technology.

## Discussion

### Food resources

The core area around the oviposition site was where the first or last eggs were laid in the habitat. In KNR, more eggs were laid in the core habitat (87 sites) than in the marginal habitat (four sites) [30]. Unlike all previous studies in which *G*. *intestinalis* was the predominant botfly species, followed by *G*. *nasalis* (both species have worldwide distributions), these species were the fourth and fifth most common in KNR (Liu, unpublished). This difference might be related to food availability, the surrounding environment, and distribution across habitats.

It is well known that botflies are considered as relatively stenothermal parasites and matured females need warm, sunny, and windless weather. There is no doubt that a higher extensiveness of *Gasterophilus* sp. infection is reported in warmer countries [16, 23, 38, 42]. Moreover, the extensiveness of the infection may be also influenced by the age and sex of examined horses [23]. However, results reported by several authors are not consistent [36, 38, 45]. The climate in northern and central Kazakhstan is continental with hot summers and very cold winters and similar to KNR in China where one finds a typical Gobi Desert habitat, subject to relatively severe climate differences, with a long, cold winter and hot, dry summer [13, 23]. The KNR area is generally considered to follow non-equilibrium dynamics in biomass production, and as a consequence, ungulate population fluctuations are driven by the amount and timing of rainfall events. *G*. *intestinalis* and *G*. *nasalis* may not be adapted to this environment. In addition, if the egg shell becomes too dry, the hatching larvae might not be able to exit the shell [53]. *G*. *pecorum* female flies lay their eggs on grass and hay [61]. Most of the eggs were laid on *S*. *capillata*, which is the main food of equines in KNR. The *G*. *pecorum* egg-laying habitat was very similar to the wild horse forging habitat [29]. Therefore, the botfly behaviour increases the chance that the eggs are eaten by horses and risk of future spread.

### Water resources

Oviposition sites may also be related to water resources [8]. Water is a key resource for most large-bodied mammals in arid lands [21, 46]. Although some bovids can survive without access to open water, like the Arabian Oryx (*Oryx leucoryx*) [37] and Addax antelope (*Addax nasomaculatus*) [22], most large ungulates need to drink regularly [20]. Przewalski’s horses seem to drink daily [49]. Asiatic wild asses are primarily adapted to arid desert steppes and semi-deserts and can venture farther from water and are better able to exploit resources that vary spatiotemporally. This suggests that zoo-born Przewalski’s horses are able to adapt their spatial use to differences in the local habitat conditions [24]. We found that 72.53% of the oviposition sites were less than 20 m from paths used by the wild horses. However, range contraction around water sources [25] and the shortest distance to the nearest water source during the summer months show that availability of water is an important factor determining space and habitat use for both species. Therefore, if the oviposition site for *G*. *pecorum* is close to a water resource, the chance of host infection will increase. This likely supports the survival of *G*. *pecorum*.

### Other factors related to oviposition site selection

Female *G*. *intestinalis* deposit their eggs mainly on the distal forelegs of the host and occasionally on the hindlegs and belly. Females of *G*. *nasalis*, *G. haemorrhoidalis*, *G. inermis*, and *G. nigricornis* deposit their eggs on the head, near the mouth. Unlike the other species, female *G*. *pecorum* lay their eggs on grass and hay [61]. In agreement with previous studies, Wang and Xu [56] and Pilo et al. [41] confirmed that female *G*. *pecorum* lay their eggs in pasture. By contrast, Cogley and Cogley [14] reported that *G*. *intestinalis* female flies lay eggs on the coat of horses. Coat colour also would seem to influence the number of eggs laid and adult flies have been shown to prefer darker horses and nearly ignored the lighter horse that was grazing in the immediate vicinity, whereas according to Pandey et al. [40] and Brocard and Pfister [7] there is no preference or attraction by *Gasterophilus* flies to any particular colour of horse.

We found that female *G*. *pecorum* laid their eggs on *S*. *capillata* in KNR and with about 2–4 eggs adhered to each plant. This egg scattering behaviour is advantageous for infecting the host. Chereshnev [11] reported that roughly 1300–2400 *G*. *pecorum* eggs can infest an entire ranch. The trend of oviposition of different *Gasterophilus* spp. (other than *G. pecorum*) is to lay eggs at different sites of the horse’s coat [41] that match the wild horse habitat, which may be an adaptive behaviour in the co-evolution of host (horse) and parasite (*Gasterophilus*). This behaviour of *G*. *pecorum* provides better opportunities for success in infesting a suitable host after hatching, and reduces the chance of offspring dying for lack of a host [39]. Asiatic wild asses are potentially exposed to a higher risk of parasite re-infection due to their temporal aggregation in very large groups.

### Synthesis and applications

It is important to study how and why eggs are distributed across potential hosts by ovipositing females. Such studies have historically been dominated by the “preference-performance problem” [32, 50], under the assumption that host preference has evolved to assure maximum offspring performance. An ovipositing female is faced with some intricate problems. She has to process sufficient information to locate and evaluate potential locations for oviposition, and she has to do this quickly and accurately [3]. This is in itself a complex, resource-intensive task, especially when the range of possible oviposition locations or prey types increases [3, 17]. This hypothesis appears to be verified by *G*. *pecorum*, because the flies choose only *S*. *capillata* for laying eggs among numerous plant species.
